# Determinants of echocardiographic epicardial adipose tissue in a general middle-aged population - The Cardiovascular Risk in Young Finns Study

**DOI:** 10.1038/s41598-024-61727-7

**Published:** 2024-05-25

**Authors:** Behnoush Gustafsson, Suvi P. Rovio, Saku Ruohonen, Nina Hutri-Kähönen, Mika Kähönen, Jorma S. A. Viikari, Katja Pahkala, Olli T. Raitakari

**Affiliations:** 1 Research Center of Applied and Preventive Cardiovascular Medicine, University of Turku, Turku, Finland; 2 Center for Population Health Research, University of Turku and Turku University Hospital, Turku, Finland; 3 Orion Pharma, Turku, Finland; 4 Department of Pediatrics, Tampere University Hospital and Faculty of Medicine and Health Technology, Tampere University, Tampere, Finland; 5 Department of Clinical Physiology, Tampere University Hospital and Faculty of Medicine and Health Technology, Tampere University, Tampere, Finland; 6 Department of Medicine, University of Turku, Turku, Finland; 7 Division of Medicine, Turku University Hospital, Turku, Finland; 8 Department of Clinical Physiology and Nuclear Medicine, Turku University Hospital, Turku, Finland; 9 Paavo Nurmi Centre, Unit for Health and Physical Activity, University of Turku, Turku, Finland; 10 Department of Public Health, University of Turku and Turku University Hospital, Turku, Finland

**Keywords:** Metabolic syndrome, Metabolic diseases, Cardiovascular diseases, Endocrine system and metabolic diseases, Metabolic disorders, Endocrine system and metabolic diseases, Epidemiology, Outcomes research, Prognostic markers

## Abstract

Epicardial adipose tissue (EAT) is the cardiac visceral fat depot proposed to play a role in the etiology of various cardiovascular disease outcomes. Little is known about EAT determinants in a general population. We examined cardiometabolic, dietary, lifestyle and socioeconomic determinants of echocardiograpghically measured EAT in early adulthood. Data on cardiometabolic, dietary, lifestyle and socioeconomic factors were collected from participants of the Cardiovascular Risk in Young Finns Study (YFS; N = 1667; age 34–49 years). EAT thickness was measured from parasternal long axis echocardiograms. Multivariable regression analysis was used to study potential EAT determinants. Possible effect modification of sex was addressed. Mean EAT thickness was 4.07 mm (95% CI 4.00–4.17). Multivariable analysis [β indicating percentage of change in EAT(mm) per one unit increase in determinant variable] indicated female sex (β = 11.0, P < 0.0001), type 2 diabetes (β = 14.0, P = 0.02), waist circumference (cm) (β = 0.38, P < 0.0001), systolic blood pressure (mmHg) (β = 0.18, P = 0.02) and red meat intake (g/day) (β = 0.02, P = 0.05) as EAT determinants. Sex-specific analysis revealed age (year) (β = 0.59, P = 0.01), alcohol intake (drinks/day) (β = 4.69, P = 0.006), heavy drinking (yes/no) (β = 30.4, P < 0.0001) as EAT determinants in women and fruit intake (g/day) (β = −1.0, P = 0.04) in men. In the YFS cohort, waist circumference, systolic blood pressure and red meat intake were directly associated with EAT among all participants. In women, age, alcohol intake, heavy drinking and type 2 diabetes associated directly with EAT, while an inverse association was observed between fruit intake and EAT in men.

## Introduction

Epicardial adipose tissue (EAT) is the cardiac visceral fat depot on the surface of the myocardium surrounding the heart and coronary arteries with no separating fascia, thus sharing the same microcirculation with myocardium^1,2^. EAT is considered a biologically active depot possibly playing a role in the etiology of cardiovascular diseases^3^ such as coronary artery disease^4^, stroke^5^, atrial fibrillation^6^ and heart failure with preserved ejection fraction^3^. In normal physiologic condition, EAT has multifaceted cardioprotective properties such as cushioning coronary arteries against mechanical stress, thermoregulating myocardium under hypothermia^7^ and maintaining myocardial free fatty acid (FFA) homeostasis due to its high capacity of FFA uptake and oxidation^8^. However, increased EAT thickness may associate with higher cardiometabolic risk^9^ and adverse subclinical changes in cardiac structure and function^10^. Prior studies have reported associations of age^11^,obesity^12^, hyperglycemia and insulin resistance^13^, dyslipidemia^14^, high blood pressure^15^, type 2 diabetes^16^, hypertension^17,18^, and fatty liver disease^19^ with increased EAT. The links between visceral adiposity, adverse metabolic profile and higher EAT thickness have been previously documented^2,3,20^, but the majority of the prior observations are from older study populations with high rates of metabolic and cardiovascular morbidities^12^. Simultaneously, there is much less evidence on modifiable determinants of EAT from large population-based cohorts of young or middle-aged adults and data on possible sex-specific EAT determinants are scarce. Since various cardiovascular diseases prevalence and risk, body fat distribution pattern and adipose tissue metabolism are known to differ between sexes, it is plausible that there may be potential sex-specific differences in epicardial adipose tissue determinants as well as a visceral fat depot. Furthermore, while various intervention studies have reported favorable effects of dietary and lifestyle interventions on EAT^21^, the independent roles of diet, lifestyle and socioeconomic factors on EAT have been poorly examined in observational settings. We aimed to identify modifiable determinants of echocardiographically measured EAT by studying the associations of cardiometabolic, dietary, lifestyle and socioeconomic factors and EAT in a population-based cohort of adults and to further seek for possible sex-specific associations among these risk factors. We hypothesize that adverse cardiometabolic (age, abdominal obesity, fasting plasma glucose, insulin resistance, serum lipid profile, blood pressure levels), poor dietary (high consumption of red meat, low fruit and vegetables intake and high alcohol intake), poor lifestyle (smoking, physical inactivity, heavy drinking) and low socioeconomic (annual income, education) factors are associated with higher EAT in early adulthood.

## Material and methods

### Study participants

This study is a part of the ongoing multi-centre longitudinal Cardiovascular Risk in Young Finns study (YFS), focusing on cardiovascular risk factors from childhood through adulthood^22^. In 1980, a representative sample (N = 3596) of Finnish 3–18-year-old children and adolescents participated in the baseline study. Follow-up studies have been conducted in 1983, 1986, 2001, 2007 and 2011. Extensive data on cardiovascular risk factors have been collected and archived from all follow-up studies^22^. From the original cohort, 1937 individuals underwent transthoracic echocardiographic examination in the 2011 follow-up study^23^. Pregnant women (N = 9) and individuals with no/low quality echocardiographic images for EAT measurement (N = 261) were excluded from this study. In total 1667 participants (770 men vs. 897 women, age 33–49 years) provided acceptable echo images and were included. YFS has been approved by ethics committee of Turku University Hospital and written informed consent has been obtained from all participants.

### Echocardiographic measurements of EAT thickness

**Figure 1:**
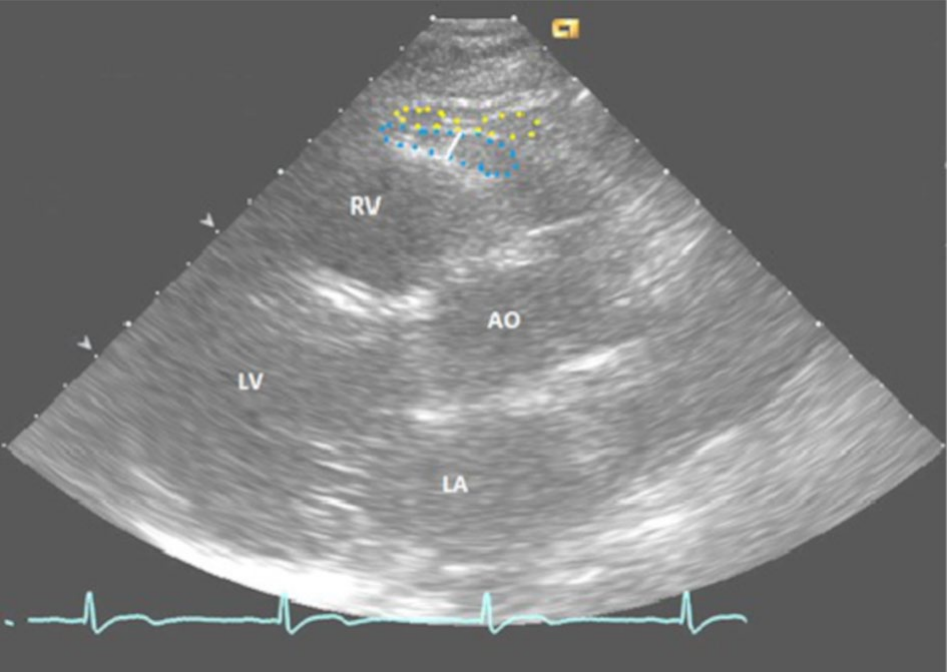
Example image of echocardiograms with visible pericardial and epicardial layer. Epicardial fat depot (blue dots). Pericardial fat depot (yellow dots). EAT thickness measured perpendicular to right ventricle at the level of aortic annulus (white line). *RV* right ventricle, *LV* left ventricle, *LA* left atrium, *AO* aorta.

Transthoracic echocardiography was performed according to the joint American and European guidelines^24^. Trained sonographers performed echocardiographic examination at five research centres using identical Siemens Acuson Sequoia 512 (Acuson Mountain view, CA) ultrasonography mainframes, equipped with 3.5 MHz scanning frequency phased-array transducers. Echocardiograms have been analysed by one observer blinded to clinical details using ComPACS 10.8.7 analysis software (Medimatic Solutions, Italy). EAT thickness was measured based on previously introduced protocol by Iacobellis et al. that has been validated using magnetic resonance imaging^25^. EAT thickness was defined as the echo free space on the right ventricle located between the outer wall of the myocardium and the visceral layer of pericardium. Because it is compressed during diastole, EAT layer is best visualized at end-systole at the midpoint of the free wall of the right ventricle and at aortic annulus level being used as an anatomic landmark^25^. Accordingly, we measured EAT thickness by manual delineation from parasternal long-axis echocardiograms perpendicularly on right ventricle at end-systole. (Fig. 1). The criteria for acceptable echo images for EAT measurement were defined as; (1) right ventricle, left ventricle, aorta and aortic annulus are clear in the image; (2) while scrolling image series, serous layer of pericardium separating epicardial fat layer from pericardial fat should be adequately visible (allowing differentiation between these distinct fat depots); (3) the signal/noise ratio should be appropriate enough to enable fat visualization (especially for very thin fat layers). A subpopulation of 50 randomly selected images was selected and re-measured to assess intra-observer variability of EAT measurements and intraclass correlation coefficient showed good reproducibility of EAT measurements (0.91, 95% CI 0.84–0.94).

### Anthropometric measurements, clinical and lifestyle characteristics

Height and waist circumference were measured and BMI was calculated as weight (kg)/height (m)^2^. Waist circumference was measured at the mid-point between lowest rib and crest ilium. Blood pressure was measured three times in sitting position by random zero sphygmomanometer with a two-minute interval between the measurements. Average of the three measurements was calculated and used in the analysis.

Data regarding daily alcohol consumption, physical activity, and smoking were queried. Daily alcohol consumption was acquired using standardized questionnaire and the number of drinks/day (one dose equal to 12 gr pure ethanol) was calculated by dividing total number of consumed doses based on self-reported weekly consumption of cans (1/3 L) of beer, number of wine glasses and strong alcohol shots (4 cL) in a week divided by seven. Heavy drinking was defined as consumption of > 6 doses of alcohol at one time and at least twice a week^26^. Leisure time physical activity index was calculated applying data on physical activity intensity, duration and frequency during leisure time (range 5–15)^27^. Smoking was classified as daily smokers versus never/rarely smokers. Dietary consumption of fruits, vegetables and red meat (grams/day) was assessed using a self-administered food frequency questionnaire, developed and validated by Finnish Institute for Health and Welfare^28^. Average annual income and educational years were used as indicators of socioeconomic status (SES). Annual income strata were classified on a 13-point scale from 1 (< 5000 €) to 13 (> 60,000 €) and educational years ranged from 10 to 22 years. Participants were classified as having type 2 diabetes if the fasting serum glucose was ≥ 7.0 mmol/L or if they received oral hypoglycaemic drugs and/or insulin, if they were diagnosed by a physician for type 2 diabetes or diagnosis was confirmed from patient data registry of Social Insurance Institution of Finland^29^. Participants were considered having hypertension if they had systolic blood pressure ≥ 140 mmHg, diastolic blood pressure ≥ 90 mmHg or they were under antihypertensive medications^30^. Data on cardiovascular outcomes (N = 45) such as coronary artery disease, peripheral artery disease and atherosclerotic cerebrovascular disease, was obtained from national data registries and the Care Register for Health Care and the National Death Index^31^. Data on lipid-lowering (N = 62) and antihypertensive (N = 163) medication was self-reported while register-based data on diabetes medication (N = 22) was used.

### Biochemical analysis

Venous blood samples were drawn after overnight fast and serum was separated, aliquoted, and stored at -70 °C. Fasting serum glucose, total cholesterol, high-density lipoprotein cholesterol (HDL-C), triglycerides, alanine transaminase (ALAT) and aspartate aminotransferase (ASAT) were measured by standard enzymatic methods^22^. Low-density lipoprotein cholesterol (LDL-C) was assessed using the Friedewald’s formula^32^. Serum apolipoprotein A1 (ApoA1), apolipoprotein B (ApoB), and C-reactive protein (CRP) were analysed using a turbidimetric immunoassay kit^26^. Serum insulin concentrations were determined with microparticle immunoassay and glycated haemoglobin A1c (HbA1c) with immunoturbidimetric method. HOMA-IR index was calculated by homeostasis model assessment of insulin resistance; (insulin × fasting glucose)/22.5. Detailed description of the methods has been reported earlier^22^.

### Statistical analysis

Cardiometabolic, lifestyle and socioeconomic variables are described as means across EAT quartiles. Continuous variables are reported as mean and standard deviation, and dichotomous/categorical variables as frequencies and percentages. Normal distribution of the variables was evaluated visually and analysed using Kolmogorov–Smirnov test. Non-normally distributed variables (BMI, waist circumference, serum HDL-C, ApoA1, ApoB, insulin, HOMA-IR, CRP and vegetable consumption) were logarithmically transformed. Student’s t-test was used for normally distributed variables, while non-parametric Mann–Whitney U-test was used for variables that remained skewed even after logarithmic transformation.

**Figure 2:**
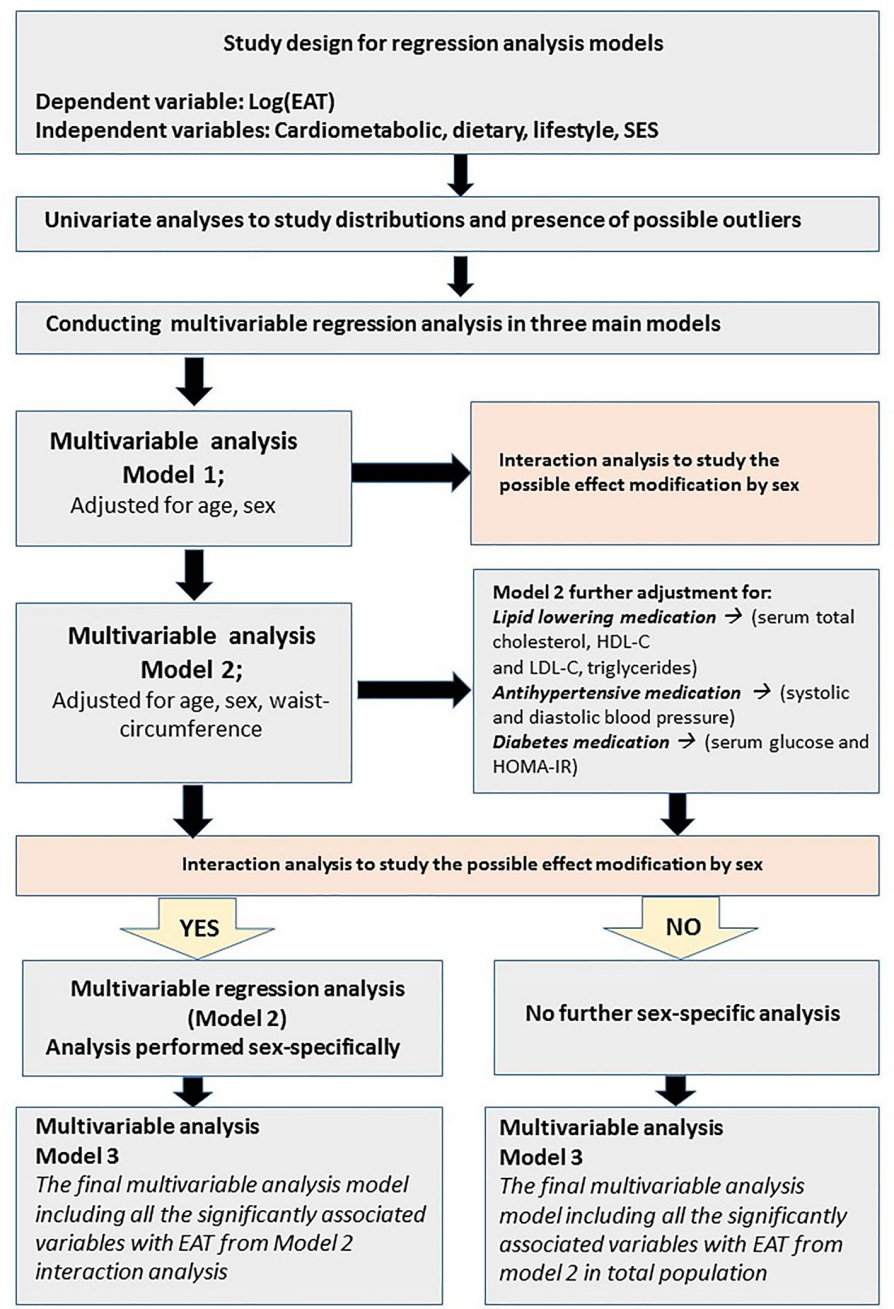
Statistical analysis depicting multivariable regression analysis models.

Linear regression models were conducted with EAT thickness as a dependent variable and each separate risk factor as an independent variable. For each risk factor, the analyses were first adjusted for age and sex (Model 1), and subsequently additionally for waist circumference (Model 2; model for BMI not adjusted for waist circumference). Additionally, we further adjusted the models related to (1) lipid profile (serum total cholesterol, HDL-C and LDL-C, triglycerides) for lipid lowering medication, (2) systolic and diastolic blood pressure for antihypertensive medication, and (3) serum glucose and HOMA-IR for diabetes medication. The distribution of the residual’s of EAT thickness (i.e. the dependent variable) was skewed for almost all of the independent variables. Based on this observation, we decided to transform the EAT variable and to conduct the multivariable regression analysis with the transformed EAT for all exposure variables. Thus, logarithmically transformed EAT was used, and the β estimates were transformed back to the original scale to ease interpretation. After the back-transformation, the estimates were interpreted as the percentage change of EAT thickness (mm) per one unit of increase in each risk factor. Finally, a multivariable regression model including all variables showing association with EAT in Model 2 were introduced to the same statistical model (Model 3; due to correlation between heavy drinking and alcohol intake, separate models were conducted). Sensitivity analyses were conducted for the Model 3 excluding the participants with cardiovascular outcomes (coronary artery disease, peripheral artery disease and atherosclerotic cerebrovascular disease). To investigate the possible effect modification by sex, we entered a multiplicative interaction term (sex × determinant variable) in the multivariable models. For interaction analysis, we decided to choose a more liberal P-value (P < 0.20), in order to assure that all possible sex-specific associations would be studied. Of note, fruit intake was the only variable included in the sex-specific analyses with p-value between 0.05 and 0.20. Those variables for which any possible effect modification was observed, sex-stratified multivariable analyses were conducted (Fig. 2). To eliminate the chance of random findings due to multiple testing, Benjamini–Hochberg correction was applied for all multivariable models. False discovery rate was set as 0.10 and considered appropriate based on the number of tests performed in this analysis (N = 30). Benjamini–Hochberg critical value has been calculated as 0.10× (i/m), where i is the rank, m is the total number of tests performed, and 0.10 is the chosen false discovery rate. The parameters original P-values were ordered from smallest to largest, the smallest value was ranked as i = 1, then second smallest ranked as i = 2, etc. Each individual P-value was further compared to its calculated Benjamini–Hochberg critical value. The largest P-value, which was less than its correspondent Benjamini–Hochberg critical value and all of the values smaller than it was selected and recognized as statistically significant. SAS 9.4 software version was used for all statistical analysis and all available data were used. The level of statistical significance was set as P < 0.05.

## Results

**Figure 3:**
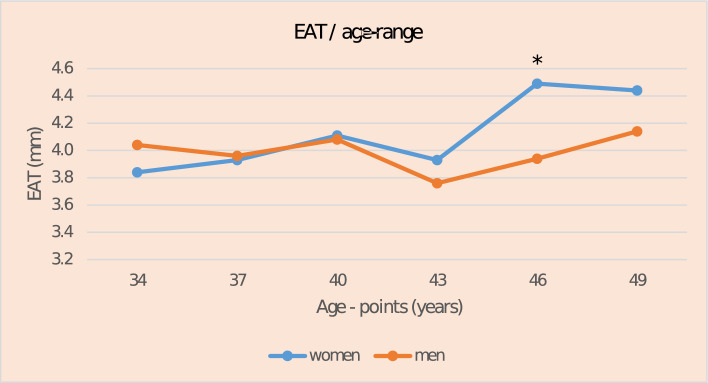
Mean EAT thickness across different age-points in men and women; Age × EAT interaction term: P = 0.002, “*”P <0 .01.

Characteristics of the study population and descriptive values of the determinants across EAT thickness quartiles are shown in Table 1 and Supplementary Tables S1-S2. Mean age was 41.9 years with no difference between men and women (41.9 ± 5.0 vs. 42.0 ± 5.0, P = 0.69). Mean EAT thickness was 4.07 mm (range 1.2–11.3 mm) and no difference was observed between women and men (Table 1) Direct association between age and EAT thickness was observed among all participants (β = 0.59 (% change in EAT per one year aging), P = 0.0009). In sex-stratified analyses, the direct association between age and EAT thickness was observed only in women (women: β = 1.00, P < 0.0001 vs. men: β = 0.03, P = 0.89, interaction term age × sex P = 0.003). (Tables 2, Model 1) These associations were similar after additional adjustment for waist circumference (Tables 2, Model 2; women: β = 0.82, P = 0.0007 vs. men: β = -1.00, P = 0.52, interaction term age × sex P = 0.002). In order to gain better understanding on the possible sex-specific mean EAT thickness changes by age, we explored the mean EAT thicknesses in different age-points separately for men and women. (Fig. 3) At younger ages (< 40 years) mean EAT thickness is higher in men with a gradual increase by age in both sexes (Fig. 3). At age 46 years, a significant (P = 0.002) abrupt increase in mean EAT thickness is observed only in women compared to men.

**Table 1:** Clinical characteristics of the study population.

	Total (N = 1667)	Male (N = 770)	Female (N = 897)	P-value
Age (yrs)	41.90 ± 4.98	41.85 ± 4.99	41.95 ± 4.98	0.69
EAT thickness (mm)	4.07 ± 1.53	3.99 ± 1.44	4.14 ± 1.60	0.08
BMI (kg/m^2^)	26.44 ± 5.00	26.96 ± 4.36	26.00 ± 5.45	** < 0.0001**
Waist circumference (cm)	92.30 ± 14.08	97.45 ± 12.64	87.91 ± 13.76	** <0.0001**
Total cholesterol (mmol/L)	5.19 ± 0.94	5.33 ± 1.0	5.07 ± 0.87	** <0.0001**
HDL cholesterol (mmol/L)	1.33 ± 0.33	1.20 ± 0.29	1.43 ± 0.32	** <0.0001**
LDL cholesterol (mmol/L)	3.27 ± 0.82	3.42 ± 0.88	3.14 ± 0.75	** <0.0001**
Triglycerides (mmol/L)	1.31 ± 0.89	1.59 ± 1.09	1.07 ± 0.58	** <0.0001**
ApoA1 (g/L)	1.58 ± 0.23	1.51 ± 0.21	1.65 ± 0.24	** <0.0001**
ApoB (g/L)	1.06 ± 0.28	1.16 ± 0.29	0.97 ± 0.24	** <0.0001**
Glucose (mmol/L)	5.39 ± 0.91	5.55 ± 0.84	5.26 ± 0.94	** <0.0001**
Insulin (mU/L)	9.48 ± 10.2	10.5 ± 11.9	8.5 ± 8.3	**0.0014**
HOMA-IR	2.49 ± 4.12	2.80 ± 4.56	2.21 ± 3.68	** <0.0001**
HbA1c (mmol/mol)	36.6 ± 5.16	36.9 ± 5.80	36.4 ± 4.53	**0.04**
ALAT (U/I)	17.9 ± 13.7	23.2 + 15.5	13.3 + 9.8	** <0.0001**
ASAT (U/I)	23.3 ± 12.2	26.4 ± 11.5	20.7 ± 12.2	** <0.0001**
CRP (mg/L)	1.66 ± 2.62	1.54 ± 2.57	1.77 ± 2.65	0.86
Systolic BP (mmHg)	118.4 ± 13.8	122.5 ± 13.1	114.8 ± 13.4	** <0.0001**
Diastolic BP (mmHg)	74.6 ± 10.4	77.6 ± 10.8	72.0 + 9.3	** <0.0001**
Type 2 diabetes (% , N)	3.9 (65)	1.9 (31)	2.0 (34)	0.80
Hypertension (% , N)	18.7 (311)	11.3 (187)	7.4 (124)	** <0.0001**
Lipid lowering medication (% , N)	3.9 (62)	2.7 (42)	1.2 (20)	**0.0003**
BP medication (% , N)	10.3 (163)	5.1 (81)	5.2 (82)	0.26
Diabetes medication (% , N)	1.4 (22)	0.8 (12)	0.6 (10)	0.39
Smoking (% , N)	15.1 (238)	8.1 (127)	7.0 (111)	**0.008**
PAI (range: 5–15)	9.03 ± 1.87	8.86 ± 1.87	9.17 ± 1.86	**0.0014**
Alcohol intake (drink doses/day)	0.85 ± 1.22	1.24 ± 1.53	0.52 ± 0.72	** <0.0001**
Heavy drinking (% , N)	6.40 (100)	4.6 (71)	1.8 (29)	** <0.0001**
Vegetables (g/day)	283.0 ± 184.1	252.3 ± 166.0	306.4 ± 193.0	** <0.0001**
Fruit (g/day)	165.0 ± 146.4	133.0 ± 129.0	189.4 ± 154.0	** <0.0001**
Fish (g/day)	49.30 ± 32.64	55.47 ± 35.49	44.61 ± 29.46	** <0.0001**
Red meat (g/day)	140.2 ± 80.6	181.0 ± 85.9	109.1 ± 60.0	** <0.0001**
Annual income (range: 1–13)	7.46 ± 3.06	8.43 ± 3.13	6.63 ± 2.75	** <0.0001**
Educational years (yrs)	14.91 ± 2.77	14.57 ± 2.79	15.9 ± 2.71	** <0.0001**

*EAT* epicardial adipose tissue, *BMI* body mass index, *HDL* high-density lipoproteins, *LDL* low-density lipoproteins, *ApoA1* apolipoprotein A1, *ApoB* apolipoprotein B, *HOMA-IR* homeostasis model assessment of insulin resistance, *HbA1c* glycated hemoglobin A1c, *ALAT* alanine transaminase, *ASAT* aspartate aminotransferase, *CRP* C-reactive protein, *BP* blood pressure, *PAI* physical activity index.

Significant values are in bold.

**Table 2:** Multivariable regression models of EAT and cardiometabolic, lifestyle, dietary and socioeconomic variables.

N = 1667	Model1						Model2					
	Total		Sex-specific				Total		Sex-specific			
			Men		Women				Men		Women	
	β	P	β	P	β	P	β	P	β	P	β	P
Age (yrs)	**0.59**	0.001*	0.03	0.89	**1.00**	** <0.0001**	**0.36**	0.04*	−1.00	0.52	**0.82**	**0.00**
BMI (kg/m^2^)	**1.34**	<0.0001*	**1.00**	**0.00**	**1.50**	** <0.0001**	Not applicable					
Waist circumference (cm)	**0 .53**	<0.0001*	**0.40**	** <0.0001**	**0.60**	** <0.0001**	Not applicable					
Total cholesterol ^ѱ^ (mmol/L)	1.54	0.11					0.84	0.39				
HDL cholesterol ^ѱ^ (mmol/L)	−4.00	0.24*	3.23	0.46	**− 8.00**	**0.04**	3.59	0.24				
LDL cholesterol ^ѱ^ (mmol/L)	1.65	0.14					1.15	0.31				
Triglycerides ^ѱ^ (mmol/L)	**2.30**	0.02*	0.21	0.85	**8.79**	** <0.0001**	−1.00	0.60*	−2.00	0.26	2.32	0.30
ApoA1 (g/L) ^ѱ^	−1.00	0.81*	7.95	0.21	−6.00	0.25	3.67	0.36				
ApoB (g/L) ^ѱ^	**9.13**	< 0.01*	1.60	0.72	**19.0**	**0.00**	0.95	0.79*	−4.00	0.50	5.65	0.30
Glucose (mmol/L) ^Ω^	**5.97**	<0.0001*	**3.95**	**0.03**	**8.28**	** <0.0001**	2.53	0.09*	1.40	0.53	3.71	0.07
Insulin (mU/L) ^Ω^	**0.26**	< 0.01*	0.09	0.36	**0.58**	** <0.0001**	−1.00	0.14*	**−1.00**	**0.03**	0.10	0.51
HOMA-IR ^Ω^	0.41	0.05*	0.11	0.69	**0.83**	**0.01**	−1.00	0.16*	**−1.00**	**0.02**	0.31	0.42
HbA1C (mmol/mol)	**0.38**	**0.02**					0.01	0.92				
ALAT (U/I) ^α^	**0.27**	<0.0001*	0.14	0.07	**0.52**	** <0.0001**	0.11	0.14*	0.06	0.47	0.21	0.10
ASAT (U/I) ^α^	**0.19**	** < 0.01**					0.06	0.39				
CRP (mg/L)	**1.95**	** <0.0001**					**1.05**	** < 0.01**				
Systolic BP^ν^ (mmHg)	**0.28**	** <0.0001**					**0.19**	** < 0.01**				
Diastolic BP^ν^ (mmHg)	**0.20**	0.02*	0.07	0.52	**0.35**	** < 0.01**	−1.00	0.78*	−1.00	0.74	−1.00	0.88
Type 2 diabetes (yes/no)	**18.7**	** < 0.01**					**10.4 **	**0.03**				
Hypertension (yes/no)	**6.00**	0.01*	1.06	0.73	**13.5**	** < 0.001**	1.00	0.67*	−3.00	0.36	**7.65**	**0.04**
Smoking (Daily vs. never/rarely smokers)	4.28	0.10					0.40	0.11				
PAI (range: 5–15)	**−2.00**	**0.01**					−1.00	0.20				
Alcohol (drink/day)	**2.16**	< 0.01*	1.36	0.12	**4.90**	** < 0.01**	**1.73 **	0.02*	0.97	0.26	**4.70**	**0.00**
Heavy drinking (yes/no)	**8.76**	0.02*	−2.0	0.74	**37.0**	** <0.0001**	7.14	0.06*	−3.00	0.62	**31.1**	** <0.0001**
Vegetables (g/day)	−1.00	0.72					0.00	0.91				
Fruits (g/day)	**−1.00**	0.02*	**−1.0**	**0.01**	−1.00	0.31	−1.00	0.08*	**−1.00**	**0.04**	−1.00	0.48
Fish (g/day)	0.00	0.83					0.01	0.67				
Red meat (g/day)	**0.04**	** < 0.01**					**0.03 **	**0.03**				
Annual income index (range 1–13)	**−1.00**	0.04*	−1.0	0.78	**−2.00**	** < 0.01**	−1.00	0.17				
Educational years (yrs)	−1.00	0.11*	−1.0	0.99	**−2.00**	**0.02**	−1.00	0.62*	0.34	0.48	−1.00	0.13

Model 1: age and sex adjusted model.

Model 2: age, sex and waist circumference adjusted model.

*BMI* body mass index, *HDL* high density lipoproteins, *LDL* low density lipoproteins, *ApoA1* apolipoprotein A1, *ApoB* apolipoprotein B, *HOMA-IR* homeostasis model assessment of insulin resistance, *ALAT* alanine transaminase, *ASAT* aspartate aminotransferase, *CRP* C-reactive protein, *BP* blood pressure, *PAI* physical activity index.

*Significant interaction between sex*determinant variable P < 0.20.

^β^Indicates the percentage change in EAT thickness (mm) per one unit increase in each variable.

^ѱ^Adjusted for lipid lowering medication in model 2.

^Ω^Adjusted for diabetes medication in model 2.

^α^Adjusted for alcohol consumption in model 2.

^ν^Adjusted for antihypertensive medications in model 2.

Significant values are in bold.

**Table 3:** Multivariable linear regression analysis of EAT determinants after Benjamini–Hochberg correction for multiple testing.

N = 1667	Model1						Model2					
	Total		Sex-specific				Total		Sex-specific			
			Men		Women				Men		Women	
	β	P	Β	P	β	P	β	P	β	P	β	P
Age (yrs)	**0.59**	0.001*	0.03	0.89	**1.00**	** <0.0001**	0.36	NS*	−1.00	0.52	**0.82**	**0.00**
BMI (kg/m^2^)	**1.34**	<0.0001*	**1.00**	**0.00**	**1.50**	** <0.0001**	Not applicable					
Waist circumference (cm)	**0 .53**	<0.0001*	**0.40**	** <0.0001**	**0.60**	** <0.0001**	Not applicable					
Total cholesterol ^ѱ^ (mmol/L)	1.54	0.11					0.84	0.39				
HDL cholesterol ^ѱ^ (mmol/L)	−4.00	0.24*	3.23	0.46	**− 8.00**	**0.04**	3.59	0.24				
LDL cholesterol ^ѱ^ (mmol/L)	1.65	0.14					1.15	0.31				
Triglycerides ^ѱ^ (mmol/L)	**2.30**	0.02*	0.21	0.85	**8.79**	** <0.0001**	−1.00	0.60*	−2.00	0.26	2.32	0.30
ApoA1 (g/L) ^ѱ^	−1.00	0.81*	7.95	0.21	−6.00	0.25	3.67	0.36				
ApoB (g/L) ^ѱ^	**9.13**	< 0.01*	1.60	0.72	**19.0**	**0.00**	0.95	0.79*	−4.00	0.50	5.65	0.30
Glucose (mmol/L) ^Ω^	**5.97**	<0.0001*	3.95	NS	**8.28**	** <0.0001**	2.53	0.09*	1.40	0.53	3.71	0.07
Insulin (mU/L) ^Ω^	**0.26**	< 0.01*	0.09	0.36	**0.58**	** <0.0001**	−1.00	0.14*	−1.00	NS	0.10	0.51
HOMA−IR ^Ω^	0.41	0.05*	0.11	0.69	**0.83**	**0.01**	−1.00	0.16*	**−1.00**	**0.02**	0.31	0.42
HbA1C (mmol/mol)	**0.38**	**0.02**					0.01	0.92				
ALAT (U/I) ^α^	**0.27**	<0.0001*	0.14	0.07	**0.52**	** <0.0001**	0.11	0.14*	0.06	0.47	0.21	0.10
ASAT (U/I) ^α^	**0.19**	** < 0.01**					0.06	0.39				
CRP (mg/L)	**1.95**	** <0.0001**					**1.05**	** < 0.01**				
Systolic BP^ν^ (mmHg)	**0.28**	** <0.0001**					**0.19**	** < 0.01**				
Diastolic BP^ν^ (mmHg)	**0.20**	0.02*	0.07	0.52	**0.35**	** < 0.001**	−1.00	0.78*	−1.00	0.74	−1.00	0.88
Type 2 diabetes (yes/no)	**18.7**	** < 0.01**					10.4	NS				
Hypertension (yes/no)	**6.00**	0.01*	1.06	0.73	**13.5**	** < 0.001**	1.00	0.67*	−3.00	0.36	7.65	NS
Smoking (daily vs. never/rarely smokers)	4.28	0.10					0.40	0.11				
PAI (range: 5–15)	**−2.00**	**0.01**					−1.00	0.20				
Alcohol (drink/day)	**2.16**	< 0.01*	1.36	0.12	**4.90**	** < 0.01**	1.73	NS*	0.97	0.26	**4.70**	**0.00**
Heavy drinking (yes/no)	**8.76**	0.02*	−2.0	0.74	**37.0**	** <0.0001**	7.14	0.06*	−3.00	0.62	**31.1**	** <0.0001**
Vegetables (g/day)	−1.00	0.72					0.00	0.91				
Fruits (g/day)	**−1.00**	0.02*	**−1.0**	**0.01**	−1.00	0.31	−1.00	0.08*	−1.00	NS	−1.00	0.48
Fish (g/day)	0.00	0.83					0.01	0.67				
Red meat (g/day)	**0.04**	** < 0.01**					0.03	NS				
Annual income index(range 1–13)	**−1.00**	0.04*	−1.0	0.78	**−2.00**	** < 0.01**	−1.00	0.17				
Educational years (yrs)	−1.00	0.11*	−1.0	0.99	**−2.00**	**0.02**	−1.00	0.62*	0.34	0.48	−1.00	0.13

Model 1: age and sex adjusted model.

Model 2: age, sex and waist circumference adjusted model.

*BMI* body mass index, *HDL* high density lipoproteins, *LDL* low density lipoproteins, *ApoA1* apolipoprotein A1, *ApoB* apolipoprotein B, *HOMA-IR* homeostasis model assessment of insulin resistance, *ALAT* alanine transaminase, *ASAT* aspartate aminotransferase, *CRP* C-reactive protein, *BP* blood pressure, *PAI* physical activity index.

*Significant interaction between sex*determinant variable P < 0.20.

^β^Indicates the percentage change in EAT thickness (mm) per one unit increase in each variable.

^ѱ^Adjusted for lipid lowering medication in model 2.

^Ω^Adjusted for diabetes medication in model 2.

^α^Adjusted for alcohol consumption in model 2.

^ν^Adjusted for antihypertensive medications in model 2.

*NS* not statistically significant after Benjamini–Hochberg correction for multiple testing (marked in italics).

Significant values are in bold.

Among all participants, sex, BMI, waist circumference, glucose, HbA1c, ASAT, CRP, systolic blood pressure, type 2 diabetes and red meat intake were directly, and physical activity inversely associated with EAT thickness (Tables 2, Model 1). Among women, additionally triglycerides, ApoB, insulin, HOMA-IR, ALAT, diastolic blood pressure, hypertension, alcohol intake, and heavy drinking showed a direct association with EAT thickness, while for HDL-C, educational years and annual income inverse associations were observed (Tables 2, Model 1). In men, the only additional association in Model 1 was observed for fruit intake (Tables 2, Model 1). After additional adjustment for waist circumference (Tables 2, Model 2), the direct associations of sex, age, CRP, systolic blood pressure, type 2 diabetes as well as alcohol intake and red meat consumption with EAT thickness persisted among all participants, while the other associations attenuated. Interaction analysis was performed for all exposure variables in Model 2 to seek for possible effect modification by sex. In these analyses, a possible effect modification by sex was observed for age, hypertension, alcohol intake, heavy drinking, HOMA-IR, insulin and fruit intake (P for interaction < 0.20). Therefore, for these variables we performed Model 2 analysis sex-specifically. The sex-specific analyses results suggested direct associations between age, hypertension, alcohol intake and heavy drinking and EAT thickness in women, while inverse associations between HOMA-IR, insulin, fruit intake and EAT thickness were observed in men (Tables 2, Model 2).

The variables showing associations with EAT thickness in Model 2 both among all participants and in the sex-specific analysis were subsequently included in the final multivariable linear regression model (Model 3) to study their independent associations with EAT thickness (Table 4). Among all participants, sex and type 2 diabetes were the strongest determinants of EAT; being female was associated with 11% greater EAT thickness compared to men, and participants with type 2 diabetes had 14% greater EAT thickness than those without type 2 diabetes. Additionally, waist circumference (β = 0.38, P < 0.0001), systolic blood pressure (β = 0.18, P = 0.026) and red meat consumption (β = 0.02, P = 0.05) were found to independently associate with higher EAT thickness. Sex-specific analyses also revealed associations between age (β = 0.59, P = 0.018), heavy drinking (β = 30.48, P < 0.0001) and alcohol intake (β = 4.69, P = 0.006) and EAT thickness in women, and fruit intake (β = −1.0, P = 0.04) in men (Table 4). Sensitivity analyses excluding participants with cardiovascular outcomes showed virtually similar results. Benjamini–Hochberg correction was applied for multivariable models and some alterations were observed in the initial results. (Tables 3, 5). For the total population, all the statistically significant associations initially observed in the analyses adjusted according to the Model 1 persisted after Benjamini–Hochberg correction (Table 3, Model 1). In the sex-specific analysis, the associations between BMI, waist circumference, fruit intake and EAT in men persisted after the Benjamini–Hochberg correction, while the associations of glucose and EAT diluted. In women, however, all the significant associations initially observed persisted after Benjamini–Hochberg correction (Table 3, Model 1). When the analyses for the whole population were further adjusted for age, sex and waist circumference (Table 3, Model 2), the associations of waist circumference, CRP, systolic blood pressure and EAT persisted, while the associations of age, alcohol intake, type 2 diabetes and red meat intake diluted after Benjamini–Hochberg correction. In the sex-specific analysis, the initially observed association between HOMA-IR and EAT in men persisted, while the associations for insulin and fruit intake were diluted. In women, the associations between age, alcohol intake and heavy drinking persisted while the association of hypertension with EAT was diluted after Benjamini–Hochberg correction (Table 3, Model 2).

**Table 4:** Multivariable linear regression analysis of EAT determinants (Model 3).

Total population	β-estimate	P-value
Female sex	**11.0**	** <0.0001**
Waist circumference (cm)	**0.38**	** <0.0001**
CRP (mg/L)	0.83	0.06
SBP (mmHg)	**0.18**	**0.026**
Type 2 diabetes (yes/no)	**14.0**	**0.028**
Red meat intake (g/day)	**0.02**	**0.05**

Men		
Waist circumference (cm)	**0.59**	**0.0002**
Fruit intake (g/day)	**- 0.1**	**0.04**

Women		
Age (year)	**0.60**	**0.018**
Waist circumference (cm)	**0.59**	** <0.0001**
Hypertension (yes/no)	7.10	0.06
Alcohol intake (drinks/day)	**4.69**	**0.006**
Heavy drinking (yes/no)	**30.48**	** <0.0001**

*CRP* C-reactive protein, *SBP* systolic blood pressure, *β-estimate* indicates the percentage change in EAT thickness (mm) per one unit of increase in each determinant variable.

Significant values are in bold.

**Table 5:** Multivariable linear regression analysis of EAT determinants after Benjamini–Hochberg correction for multiple testing.

Total population	β-estimate	P-value
Female sex	**11.0**	** <0.0001**
Waist circumference (cm)	**0.38**	** <0.0001**
CRP (mg/L)	0.83	0.06
SBP (mmHg)	**0.18**	**0.026**
Type 2 diabetes (yes/no)	**14.0**	**0.028**
Red meat intake (g/day)	**0.02**	**0.05**

Men		
Waist circumference (cm)	**0.59**	**0.0002**
Fruit intake (g/day)	**- 0.1**	**0.04**

Women		
Age (year)	**0.60**	**0.018**
Waist circumference (cm)	**0.59**	** <0.0001**
Hypertension (yes/no)	7.10	0.06
Alcohol intake (drinks/day)	**4.69**	**0.006**
Heavy drinking (yes/no)	**30.48**	** <0.0001**

*CRP* C-reactive protein, *SBP* systolic blood pressure, *β-estimate* indicates the percentage change in EAT thickness (mm) per one unit of increase in each determinant variable.

Significant values are in bold.

Subsequently, the Benjamini–Hochberg correction was applied to the final multivariable model (Model 3) including all the variables, which remained significant in age, sex, waist circumference adjusted model. In the total population, the initially observed associations of female sex, waist circumference, systolic blood pressure, type 2 diabetes and red meat intake with EAT persisted. In the sex specific analysis, the associations of waist circumference and fruit intake in men and the associations of age, waist circumference, heavy drinking, alcohol intake and EAT in women also persisted after Benjamini–Hochberg correction (Table 5).

## Discussion

We examined the cardiometabolic, lifestyle and dietary determinants of EAT in a young and middle-aged cohort. Our results suggest associations of female sex, type 2 diabetes, high systolic blood pressure, large waist circumference, and consumption of red meat with thicker EAT. Our finding on the higher EAT thickness among women than men is in line with previous studies^33^. However, contradictory findings also exist.^34^ The controversy between our and prior studies in the evidence on the sex-specific EAT thicknesses could be explained by differences in age, size and ethnicity of the study populations^12,35^

It is known that visceral adipose tissue (VAT) distribution differ greatly between men and women as men accumulate more fat in the upper body (android pattern) and women are more prone to accumulate fat in lower body (gynoid pattern)^36^. The ratio of abdominal subcutaneous to visceral fat decreases in both genders by increasing age. While in men VAT is known to increase gradually with age, in women an increase in VAT accumulation is reported mainly in premenopausal and menopausal transition^36,37^. The subtle significant increase in VAT in middle-aged premenopausal and menopausal women has been suggested to occur as a consequence of alterations in sex-hormones and their adverse effects on hormonal regulation of hepatic glucose/lipid metabolism^38,39^. Post-menopausal hormonal changes has been widely addressed as one of the main underlying mechanisms explaining the shift in accumulation of VAT in post-menopausal women^38,40^. Accordingly, some studies have also addressed normalization of metabolic state and reduction of abdominal VAT deposition in post-menopausal women who have undergone hormone replacement therapies^41^. These observations have addressed the pivotal role of hormonal alterations in body fat deposition preference shift in post-menopausal women. Some studies have also addressed concomitant increase in VAT, EAT as well as hepatic fat content in premenopausal and menopausal women^36^, which were further associated with lower serum concentrations of estrogen. It is hypothesized that the shift in all these visceral fat depots occurs due to lowering estrogen level and its lipolytic activity in subcutaneous fat depots (dysfunctional subcutaneous adipose tissue) leading to reduced uptake of FFA and triglycerides by subcutaneous fat, which potentially increases the deposition and infiltration of fat in visceral depots^38,42^.

Considering the notion that VAT and EAT significantly correlate with each other, our observation on female sex being associated with higher EAT thickness in middle-age could indicate the synergistic increase in both VAT and EAT that may occur concomitantly as a result of premenopausal related hormonal alterations in this specific age range, lower estrogen levels and its lipolytic activity and more adverse lipidomic/glycemic profile in middle-aged women. However, the plausibly deterministic role of some gene variants has been suggested in the literature, but to our knowledge, no consensus on this has been achieved so far^35^.

In addition to age, sex and obesity indices, we observed direct associations of glucose, insulin, liver enzymes, CRP, and blood pressure with EAT thickness suggesting that individuals with cardiometabolic risk factors may potentially have higher EAT thickness^20^. However, most of these associations were attenuated after adjusting for waist circumference^2^. Our observations are consistent with previous studies addressing attenuation of observed associations between EAT thickness and cardiometabolic risk factors after obesity measures are taken into account. It can thus be postulated that at least part of the observed associations between cardiometabolic risk factors and EAT thickness counts for the shared links between EAT and visceral obesity^14^. Importantly, the associations of waist circumference, type 2 diabetes, systolic blood pressure and EAT thickness still persisted after adjustments for a wide array of possible confounders.

Large waist circumference is a surrogate marker of intra-abdominal visceral fat accumulation^43^. Our results suggesting that waist circumference is a determinant of EAT thickness confirm previous findings on the associations of intra-abdominal visceral fat and high EAT thickness^14^. Increased intra-abdominal visceral adiposity is accompanied by elevated circulatory FFA levels, systemic inflammation and insulin resistance as well as impaired myocardial glucose-lipid metabolism^43^. These metabolic disturbances proposedly contribute to systemic fatty acid overload and subsequent increase in EAT thickness^19,43^. In a normal physiologic condition, EAT serves as a local energy source channelling FFAs directly to myocardium and also as a cardioprotective fat reservoir to buffer FFA surplus, thus avoiding lipotoxicity of the heart^44^. However, circulatory FFA overload and systemic insulin resistance present in progressive obesity may ultimately lead to epicardial adipocytes’ fatty acid saturation, which further triggers their hypertrophic, hypoxic and aggravated inflammatory state^3^.

We found that type 2 diabetes was independently associated with higher EAT. This result confirm previous findings on increased EAT thickness in type 2 diabetes and suggests its direct associations with indices of impaired glucose tolerance and insulin resistance^12,16^. Myocardial insulin-stimulated glucose uptake is impaired in type 2 diabetes^10^ and this condition associates with higher FFA uptake and oxidation in EAT^10^. In fact, EAT thickness has been reported to increase already in prediabetes and in the presence of subclinical insulin resistance, before the actual onset of clinical symptoms^45^. Disruptions in cardiac glucose-lipid homeostasis, systemic and myocardial insulin resistance and possible dysregulation of genes associated with lipid metabolism have been suggested as possible pathways explaining elevated EAT thickness in type 2 diabetics^45,46^.

Our finding that high systolic blood pressure is independently associated with higher EAT thickness is supported by prior findings suggesting links between high blood pressure, hypertension and increased EAT^15,47^. However, the crosstalk between obesity, cardiovascular function, blood pressure regulation and EAT is complex, multifactorial and currently poorly understood^47^. Prolonged obesity, elevated blood pressure and pre-hypertension are associated with subclinical changes in cardiac structure and function such as increased left ventricular mass, increased wall thickness and subclinical systolic and diastolic dysfunction, which will ultimately lead to higher cardiac workload and increased energy demands for cardiac contractile function^48^. In such a condition, EAT thickness is proposed to increase as an initial adaptive mechanism to these subclinical alterations facilitating energy supply to the myocardium^15^.

We also observed inverse associations of physical activity and annual income with EAT thickness, but the associations diluted after adjustment for waist circumference. Likewise, various interventional studies have previously suggested potential benefits of various exercise interventions on EAT reduction^49^. Furthermore, individuals with higher income may potentially have better access to living environments promoting healthier lifestyle (e.g. better training facilities and restricted smoking areas) and dietary habits (availability of healthier foods), which are typically accompanied also with lower prevalence of obesity^50^. However, we found red meat consumption as the only dietary determinant of EAT thickness among all participants. While the underlying mechanisms linking lower red meat intake and thinner EAT remain obscure, potential explanations may be hypothesized according the results from previous studies. Improved diet quality (based on components of a Mediterranean diet) with low red meat consumption has been associated with reduced visceral and liver fat accumulation, lower weight gain and improved lipidemic and glycemic state^50–52^. These favorable changes in adiposity levels and metabolic profile in lower red meat consumption may link with reduction in EAT and other ectopic fat depots^51,52^. However, it might also be that low red meat intake is rather a proxy of healthier diet in general rather than an independent EAT determinant.

We found also some sex-specific EAT determinants. Age, hypertension, higher alcohol intake and heavy drinking were directly associated with EAT thickness in women, while higher fruit intake was inversely associated with EAT in men. It is known that aging alters body fat distribution^35,36^. Especially in postmenopausal women, aging is associated also with altered cardiometabolic risk profile and increased visceral abdominal fat accumulation^42^. In line, late premenopausal and menopausal women have been reported to have higher EAT thickness compared to younger women^38^. It is postulated that age-related premenopausal hormonal changes may associate with concomitant accumulation of visceral abdominal and EAT depots in women^40^. In our cohort, only 18 women reported being menopausal. However, increased visceral adipose tissue accumulation has been reported in middle-aged premenopausal women much before the onset of the actual menopause^39^. In addition to the menopausal transition, sexual dimorphism of EAT gene expression and adipose tissue preferential deposition associated with aging are suggested as possible factors explaining higher EAT thickness in middle-aged women compared to men^53^.

In addition to age, we found that hypertension, alcohol intake and heavy drinking associated with higher EAT in women but not in men. Associations between hypertension and EAT thickness have been observed also previously^15,54^. Although there is no consensus on the possible underlying mechanisms linking hypertension and thicker EAT specifically in middle-aged women, some potential underlying mechanisms related to adverse effects of aging, hormonal changes and concomitant increase in abdominal visceral fat and EAT and their possible association with blood pressure regulation could be evoked^15^. Gradual increase in abdominal visceral fat along with unfavorable dyslipidemic state, decreased estrogen levels and its diminished protective vasodilatory effects accompanied with higher peripheral resistance resulting in higher cardiac workload in hypertensive women^15,54^ may all be proposed as the underlying mechanisms linking hypertension and thicker EAT in middle-aged women.

In women, alcohol intake was directly associated with EAT thickness. Thicker EAT has been reported in chronic alcohol users and the mediating role of EAT has been proposed as a prognostic marker of alcoholic heart disease in chronic alcohol consumption^55^. Although no cause-effect relationship can be derived from these observed cross-sectional associations, some direct/indirect mechanisms may be hypothesized. EAT is an organ-specific fat depot, which is associated with serum FFA concentrations. In fact, EAT has been addressed to contain highest FFA uptake and utilization among ectopic fat depots^56^. A direct mechanistic pathway hypothesizes that alcohol consumption first increases adipose tissue breakdown and leads to increased circulatory FFA concentrations. This may then induce increased FFA uptake in EAT and ultimately lead to higher EAT thickness^19^. An indirect mechanistic pathway hypothesizes that the adverse effects of alcohol consumption on metabolic dysregulations associate with increased visceral obesity, insulin resistance, systemic inflammation, increased hepatic lipogenesis, steatosis and inflammatory states, which may further trigger higher EAT accumulation due to hepatic lipid metabolism disturbances^57,58^. In addition, toxic effects of ethanol on myocardial function in chronic alcohol consumption is also proposed to amplify higher EAT accumulation as a compensatory mechanism^55^. In our data, a direct link between alcohol intake and EAT thickness was observed only in women. In premenopausal women, reducing estrogen levels and its diminished favorable effects on hormonal regulation of lipid metabolism^57,59^, is associated with more visceral and hepatic adiposity as well as more unfavorable dyslipidemic and inflammatory state^58^. It is apparent that higher consumption of alcohol may reckon additional burden on already present lipid metabolism disturbances in middle-aged women possibly via these mechanistic pathways. Furthermore, lower gastric dehydrogenase activity, lesser body water content, and smaller gesture in women is suggested to further influence e.g. lower clearance rate of alcohol in premenopausal women^60^.

Additionally, we found an association between heavy drinking and EAT in women. To our knowledge, this is the first study addressing sex-specific associations between alcohol consumption pattern and EAT. It has been previously reported that not only alcohol intake but also the intensity of alcohol consumption may contribute to higher abdominal fat accumulation although the exact underlying mechanisms are not fully understood^61^. Given the probable strong correlation between abdominal fat and EAT, heavy drinking is assumed to concomitantly link with both. In men, we observed also an association between higher fruit intake and lower EAT. A prior systematic review has addressed significant associations of increased fruit intake with reduced waist circumference and lower risk of adiposity^62^. Furthermore, an intervention study applying a Mediterranean diet addressed improvements in anthropometric and metabolic profile in terms of decreased BMI and waist circumference, decreased total cholesterol and triglycerides levels and increased HDL cholesterol levels in men as compared to women in response to increased consumption of fruits and vegetables^63^. However, in that study the observed associations attenuated after adjustment for baseline measures of the cardiometabolic factors, which suggests that poorer metabolic profile at baseline could explain the observed improvements related to higher fruit intake^63^. Furthermore, showing the specific links of fruit intake with metabolic parameters and EAT is challenging due to the fact that fruit and vegetable intake often correlates with other beneficial dietary intake components, and may thus only be a proxy for a healthy diet in general.

Moreover, if the observed associations were causal, our results would indicate that in addition to weight management and cardiometabolic profile enhancement, acquiring healthier lifestyle and dietary habits (i.e. higher physical activity, lower red meat and alcohol intake and higher fruit intake) may induce beneficial effects on EAT. However, the links between obesity, cardiometabolic, lifestyle, dietary and socioeconomic factors and EAT are complex, multifactorial and plausibly interrelated. Of note, our study has some limitations. First, there was no exact data on intra-abdominal visceral fat available. We used waist circumference as a measure of abdominal adiposity acknowledging that it does not distinctively differentiate subcutaneous from visceral abdominal fat. Therefore, we could not extensively address if the higher EAT thickness is precisely a consequence of higher intra-abdominal visceral adiposity. Second, due to the observational study design, we cannot address causality of the observed associations. Third, different study populations and varying EAT measurement methods make the comparisons between our and prior results challenging. For example, the majority of the previous studies on this topic have been conducted in older cohorts where the associations are not analyzed sex-specifically^12^. Fourth, our study population consists of white Finnish individuals, which limits the generalizability of our findings to other ethnicities. Therefore, acquiring data on reference values and determinants of EAT from different populations would be important. Fifth, echocardiographic measurement of EAT, which is mainly based on one single parameter measured from parasternal long axis view may also contain some level of uncertainty due to the user-dependence of this imaging method linked e.g. to the user’s proficiency and experience. Since there is the possibility for bias and inaccuracy that may exist in our echo-based EAT measurements due to the inherent properties of echocardiography imaging, we further tried to address the reliability of the measurement by re-measurement of a random sample of 50 images and by addressing their intraclass correlation coefficient (ICC). These two distinct measurements showed good intra-observer variability. Ultimately, regarding the questionnaire data, some bias and /or misreporting might exist. Our questionnaire data has been collected always prospectively, which reduces the possible recall bias. However, there are few limitations in our questionnaire data to be addressed. Questionnaires have been kept unmodified during the follow-ups, therefore, the possible need for data standardization has been unmet in these questionnaire data. In addition, there is a possibility of general misreporting bias in particular for lifestyle and dietary factors towards healthier lifestyle/ dietary habits.The strengths of our study is the large population-based cohort of middle-aged adults including both sexes, which minimizes the possible confounding due to aging related cardiometabolic complications.

To conclude, our observations suggest that in addition to weight management approaches, application of healthy dietary and lifestyle habits in young and middle-aged adults might help to reduce EAT. Identification of modifiable risk factors for EAT in adulthood and early midlife is crucial not only to reduce cardiac adiposity but also to promote cardiometabolic health.

### Supplementary Information

Supplementary Tables.

## Data Availability

Due to local legal restrictions, data sharing outside the study group requires a data-sharing agreement. Investigators can submit an expression of interest to the YFS Steering Group/Data Sharing Committee (olli.raitakari@utu.fi).
