# Supplementary material for: Determinants of echocardiographic epicardial adipose tissue in a general middle-aged population - The Cardiovascular Risk in Young Finns Study
Source: Sci Rep. 2024 May 25;14:11982. doi: 10.1038/s41598-024-61727-7 (PMC11127977; doi:10.1038/s41598-024-61727-7)
Supplement: Supplementary file 1 — Supplementary Tables. [file 41598_2024_61727_MOESM1_ESM.docx]

**Supplementary tables**

**Article title:**

Determinants of echocardiographic epicardial adipose tissue in a general middle-aged population-The Cardiovascular Risks in Young Finns Study

**Journal:**

Scientific reports

**Authors:** Gustafsson B^1^, Rovio SP^1,2,10^, Ruohonen S^1,3^, Hutri-Kähönen N^4^, Kähönen M^5^ , Viikari JSA^6,7^, Pahkala K^1,2,9^, Raitakari OT^1,2,8^.

^1^Research Center of Applied and Preventive Cardiovascular Medicine, University of Turku, Turku, Finland;

^2^Center for Population Health Research, University of Turku and Turku University Hospital, Turku, Finland;

^3^Orion Pharma, Turku, Finland;

^4^Department of Pediatrics, Tampere University Hospital and Faculty of Medicine and Health Technology, Tampere University, Tampere, Finland;

^5^Department of Clinical Physiology, Tampere University Hospital and Faculty of Medicine and Health Technology, Tampere University, Tampere, Finland;

^6^Department of Medicine, University of Turku, Turku, Finland;

^7^Division of Medicine, Turku University Hospital, Turku, Finland;

^8^Department of Clinical Physiology and Nuclear Medicine, Turku University Hospital, Turku, Finland;

^9^Paavo Nurmi Centre, Unit for Health and Physical Activity, University of Turku, Turku, Finland

^10^Department of Public Health, University of Turku and Turku University Hospital, Turku, Finland

**Corresponding author:**

Behnoush Gustafsson, Research Center of Applied and Preventive Cardiovascular Medicine, University of Turku, Turku, Finland and Centre for Population Health Research, University of Turku and Turku University Hospital, Turku, Finland

Email:behfor@utu.fi

ORCID: 0000-0002-9211-1853

**Table S1: Distribution of cardiometabolic variables across the EAT quartiles**

| **Variables** | **Total**  **N=1667**  **770(m)/897(w)** | **Quartile 1**  **N= 409**  **197(m)/212 (w)** | **Quartile 2**  **N=394**  **177 (m)/217(w)** | **Quartile 3**  **N=442**  **215(m)/227(w)** | **Quartile 4**  **N=422**  **181 (m)/241(w)** |
| --- | --- | --- | --- | --- | --- |
| **EAT (mm)** | 4.0 + 1.5 | 2.4 + 0.4 | 3.3 + 0.2 | 4.2 + 0.3 | 6.1 + 1.2 |
| **Age (yrs)** | 41.9 + 4.9 | 41.4 + 4.8 | 41.6 + 5.0 | 42.0 + 4.9 | 42.4 + 5.0 |
| **BMI (kg/m^2^)** | 26.4 + 5.0 | 25.4 + 4.5 | 25.7 + 4.4 | 26.7 + 4.9 | 27.7 + 5.6 |
| **Waist (cm)** | 92.3 + 14.0 | 89.6 + 13.7 | 90.4 + 13.2 | 93.3 + 13.4 | 95.5 + 15.0 |
| **Total cholesterol**  **(mmol/L)** | 5.19 + 0.94 | 5.14 + 0.93 | 5.15 + 0.88 | 5.21 + 0.97 | 5.24 + 0.96 |
| **HDL cholesterol (mmol/L)** | 1.33 + 0.33 | 1.33 + 0.37 | 1.34 + 0.30 | 1.31 + 0.31 | 1.33 + 0.34 |
| **LDL cholesterol (mmol/L)** | 3.27 + 0.82 | 3.21 + 0.77 | 3.25 + 0.78 | 3.30 + 0.87 | 3.29 + 0.86 |
| **Triglycerides**  **(mmol/L)** | 1.31 + 0.89 | 1.30 + 0.90 | 1.22 + 0.70 | 1.32 + 1.04 | 1.39 + 0.87 |
| **ApoA1 (gr/L)** | 1.58 + 0.23 | 1.58 + 0.25 | 1.59 + 0.22 | 1.57 + 0.22 | 1.60 + 0.24 |
| **ApoB (gr/L)** | 1.06 + 0.28 | 1.04 + 0.27 | 1.04 + 0.27 | 1.06 + 0.29 | 1.08 + 0.28 |
| **Glucose (mmol/L)** | 5.36 + 0.69 | 5.29 + 0.56 | 5.31 + 0.61 | 5.38 + 0.69 | 5.47 + 0.84 |
| **Insulin (mU/L)** | 9.48 + 10.2 | 8.70 + 9.0 | 9.26 + 13.0 | 9.64 + 9.6 | 10.2 + 8.7 |
| **Homa index** | 2.49 + 4.12 | 2.18 + 3.35 | 2.48 + 5.49 | 2.62 + 4.42 | 2.64 + 2.78 |
| **HbA1C (mmol/mol)** | 36.6 + 5.16 | 36.2 + 3.66 | 36.7 + 6.11 | 36.6 + 5.03 | 37.0 + 5.57 |
| **ALAT (U/I)** | 17.9 + 13.7 | 16.7 + 13.2 | 16.9 + 13.5 | 17.9 + 13.9 | 19.7 + 13.9 |
| **ASAT (U/I)** | 23.3 + 12.2 | 22.6 + 10.3 | 22.6 + 9.3 | 23.8 + 15.2 | 24.3 + 12.7 |
| **CRP (mg/L)** | 1.66 + 2.62 | 1.29 + 1.84 | 1.49 + 2.24 | 1.59 + 2.64 | 2.25 + 3.36 |
| **Systolic BP (mmHg)** | 118.4 + 13.8 | 116.8 + 13.9 | 117.1 +13.2 | 119.0 +13.9 | 120.5 + 14.0 |
| **Diastolic BP(mmHg)** | 74.6 + 10.4 | 73.7 + 11.0 | 74.1 + 9.8 | 75.0 + 10.1 | 75.6 + 10.4 |
| **Type 2 diabetes**  **(% , N)** | 3.9 (65 ) | 10.8 (7) | 16.9 (11) | 27.7 (18) | 44.6 (29) |
| **Hypertension (% , N)** | 18.7 (311) | 22.2 (69) | 19.6 (61) | 28.6 (89) | 29.6 (92) |

EAT: Epicardial adipose tissue, BMI: body mass index, HDL: High density lipoproteins, LDL: Low density lipoproteins,

ApoA1:apo-lipoprotein A1, ApoB: Apolipoprotein B, HOMA: homeostasis model assessment of insulin resistance,

HbA1C: glycated hemoglobin A1c, ALAT: alanine transaminase, ASAT :aspartate aminotransferase, CRP: c-reactive protein,

BP: blood pressure

**Table S2: Distribution of lifestyle, dietary and socioeconomic variables across EAT quartiles**

| **N= 1667** | **770 (m)/897(w)** | **Quartile 1**  **N= 409**  **197(m)/212 (w)** | **Quartile 2**  **N=394**  **177 (m)/217(w)** | **Quartile 3**  **N=442**  **215(m)/227(w)** | **Quartile 4**  **N=422**  **181 (m)/241(w)** |
| --- | --- | --- | --- | --- | --- |
| **Smoking (% , N)** | 15.1 (238) | 3.3 (52) | 3.4 (53) | 4.0 (64) | 4.4 (69) |
| **Physical activity index** | 9.03 + 1.87 | 9.16 + 1.75 | 9.05 + 1.92 | 9.08 + 1.92 | 8.82 + 1.87 |
| **Alcohol(drinks/day)** | 0.85 + 1.22 | 0.75 + 1.05 | 0.82 + 1.15 | 0.89 + 1.21 | 0.93 + 1.42 |
| **Heavy drinking**  **(% , N)** | 6.4 (100) | 18.0 (18) | 25.0 (25) | 26.0 (26) | 31.0 (31) |
| **Vegetables (gr/day)** | 283.2 + 184.1 | 280.5 + 187.4 | 276.3 + 196.9 | 296.7 + 187.5 | 278.1 + 162.7 |
| **Fruit (gr/day)** | 165.0 + 146.4 | 179.2 + 153.0 | 159.2 + 143.3 | 162.3 + 131.2 | 159.6 + 157.3 |
| **Red meat (gr/day)** | 140.2 + 80.6 | 136.1 + 77.1 | 133.8 + 71.6 | 142.6 + 88.3 | 148.2 + 83.8 |
| **Income index** | 7.46 + 3.06 | 7.67 + 3.06 | 7.45 + 3.02 | 7.58 + 3.05 | 7.13 + 3.10 |
| **Educational years** | 14.9 + 2.77 | 15.0 + 2.85 | 15.0 + 2.85 | 14.8 + 2.70 | 14.7 + 2.66 |
